# Development of a method to measure protein diffusivities in hyaluronic acid solutions based on a UV–vis spectrophotometer

**DOI:** 10.1016/j.xphs.2026.104507

**Published:** 2026-09-18

**Authors:** Hao Lou, Meng-Juan Pang, Benjamin Southard, Hashim M. Reza

**Affiliations:** a Department of Pharmaceutical Chemistry, The University of Kansas, Lawrence, KS, USA; b Biopharmaceutical Innovation & Optimization Center, The University of Kansas, Lawrence, KS, USA

**Keywords:** Diffusion coefficient, 3D printing, Mass transfer, UV–vis spectroscopy, Fick’s laws of diffusion, Protein, Polymer

## Abstract

Protein diffusion within hyaluronic acid (HA)-rich extracellular matrices can be important for subcutaneous and intravitreal routes of administration. Current techniques for measuring protein diffusivities in HA solutions often require specialized instrumentation and/or complex experimental setups, hindering their widespread application in pharmaceutical laboratories. In this study, by modifying our previous method, we developed a simple, material-sparing approach to determine protein diffusion coefficients in HA solutions using a conventional UV–vis spectrophotometer. By installing a 3D-printed cover with an open slit at the front of a standard UV cuvette, the concentration of a model protein (ovalbumin) at the slit could be measured over time. The effective diffusion coefficient was then calculated from the concentration-time profile using both analytical and numerical methods (the finite difference and finite element methods). As a result, all three methods adequately described the experimental data, with average R^2^ values of approximately 0.99 and comparable diffusion coefficients. Increasing the concentration of high-molecular-weight HA (e.g., 144 kDa and above) reduced ovalbumin diffusivity to a similar degree, independent of HA molecular weights, whereas low-molecular-weight HA caused little inhibition and, under some concentrations, slightly increased protein diffusion. These findings demonstrate that the proposed UV–vis-based method provides an accessible and reliable approach for measuring protein diffusion in HA-containing biological and pharmaceutical matrices.

## Introduction

Hyaluronic acid (HA), a linear polymer composed of repeating disaccharide units of D-glucuronic acid and N-acetyl-D-glucosamine linked by β–1,4 glycosidic bonds, is found extensively and ubiquitously within the extracellular matrix of various organs and tissues in humans and other vertebrates. HA plays vital physiological roles, including general functions such as maintaining tissue hydration and organizing the extracellular matrix, as well as more specific roles during embryonic development, wound healing, carcinogenesis, aging, ROS elimination, and inflammation regulation.^1–6^ Furthermore, native HA and HA-based derivatives have been developed into products, demonstrating a wide range of medical and cosmetic applications, including osteoarthritis and cartilage regeneration, skin care, ocular treatment, wound healing, as scaffolds for tissue engineering, and as vehicles for subcutaneous/intravitreal/oral/transdermal drug delivery.^1,2,5–9^ For subcutaneous and intravitreal administration of macromolecule-based drugs, HA can act as an obstacle and/or interact strongly with drugs, significantly affecting protein transport within the extracellular matrix.^10,11^ For in vitro drug product development and testing, researchers developed artificial HA-containing media to simulate body interstitial fluids.^12^

In this study, we focus on the diffusion of macromolecules, such as proteins, in HA solutions to provide insights into in vivo drug transport and distribution and to develop a straightforward method for measuring their diffusion coefficients. To analyze the diffusion of molecules and tracer particles in polymer solutions from the diluted state to the concentrated or melt state, several techniques have been developed, including forced Rayleigh scattering,^13,14^ dynamic light scattering (DLS),^15^ pulsed field gradient NMR (PFG-NMR),^14^ fluorescence recovery after photobleaching (FRAP),^16^ X-ray photon correlation spectroscopy,^17^ and fluorescence correlation spectroscopy (FCS).^18,19^ Furthermore, Ladisch et al. recently developed a method using a custom-designed chamber and a commercially available gel imaging scanner to measure protein diffusion within HA matrices (e.g., at 10 and 20 mg/mL) by analyzing images of concentration distributions derived from the protein’s intrinsic fluorescence over time.^20–24^

It is noted that many of the above techniques require instruments that many laboratories do not have access to, and/or require significant effort for instrument setup and qualification. Here, we aim to develop a simple, material-sparing method that can be employed by most laboratories. The present work builds on our previous work, using a UV–vis spectrophotometer installed with a custom-designed cuvette (achieved by attaching a 3D-printed cover with an open slit to a standard UV cuvette) to determine diffusion coefficients of small molecules and proteins in non-viscous aqueous media.^25^ In this study, we modified the sample-injection method to better accommodate measurements in more viscous media (e.g., HA solutions), followed by method qualification, and then applied it to investigate protein diffusivities in different HA solutions.

## Materials and methods

### Materials

Ovalbumin (OVA) from chicken egg white in lyophilized powder form and all salts used for medium preparation were purchased from Sigma-Aldrich (St. Louis, MO). Sodium hyaluronate (NaHA), which dissociates into the anionic form of HA at pH 7.4 with six different molecular weight (MW) ranges (the reported average MW listed on the COAs: 4.7, 42, 144, 337, 690, and 1670 kDa), was purchased from Lifecore Biomedical (Chaska, MN). For simplicity, they are referred to as HA4.7 K, HA42K, HA144K, HA337K, HA690K, and HA1670K below. All solvents used in this study were of analytical grade.

### Cuvette design

The cuvette was designed and built using our previously established method with some modifications. As shown in Fig. 1(a), first, a custom-designed cover with an open slit was shielded with aluminum foil (leaving the slit open) and secured with double-sided tape on both sides, then attached to the front of a Hellma^®^ Quartz absorption cuvette (volume: 3.5 mL; optical path: 10 mm). The back of the cuvette was similarly covered with aluminum foil, featuring an open window aligned with the front slit. In this study, our cover design measured 45 mm in height, 12.5 mm in width, and 1 mm in thickness; the slit itself was 1 mm high and 7 mm wide, with its lower edge positioned 7 mm from the bottom of the cover. Since the cuvette’s base is 1.5 mm thick, the lower edge of the slit sits 5.5 mm above the inner bottom of the cuvette. The covers were designed in AutoCAD (Autodesk Inc., CA, USA) and manufactured using an Ultimaker S5 3D Printer (Ultimaker B.V., NY, USA).

### Experimental procedure

A Lambda 35 UV–vis spectrophotometer (PerkinElmer, MA, USA) was used to determine the diffusion coefficients of OVA in NaHA solutions at room temperature (22 ± 1 °C), as shown by a cartoon in Fig. 1(b). Two custom-designed cuvettes, prepared using the method described in Section 2.2, were loaded into the reference and sample positions, respectively. For both cuvettes, 3 mL of NaHA solution (a preset amount of NaHA powder solubilized in PBS, pH 7.4) was added. Then, using a 50-*μ*L Hamilton^®^ microliter syringe fitted with a removable needle, 50 *μ*L of OVA solution (OVA solubilized in deuterium oxide at 50 mg/mL) was added to the bottom of the sample cuvette. The injection was performed by slowly moving the syringe horizontally along the path shown in Fig. 1(c) to help the injected liquid spread evenly. After injection, the needle was left in place at the corner of the cuvette with tape (the corner position does not affect the beam path inside the cuvette). The syringe was then carefully removed, and the cuvette was sealed with Parafilm^®^. Unlike our previous measurements conducted in low-viscosity aqueous media,^25^ two key, necessary changes were made in this study: (i) slowly and horizontally moving needle positions instead of a fixed central position for OVA solution injection; and (ii) leaving the needle inside rather than pulling it out, since removing it from the viscous HA solutions often caused experimental errors due to liquid displacement and the formation of a slow-reforming channel along its removal path. The UV absorbance was set to 280 nm, and protein concentration was calculated from a standard curve generated from known concentrations. Measurements were performed in triplicate for each medium.

### Mathematical modeling

After obtaining the concentration-versus-time profiles, the effective diffusion coefficients of OVA in NaHA solutions could be calculated using three mass-transfer-based methods listed below.

### Analytical method

The first method models drug diffusion in one dimension, as shown in Fig. 1(d), using an analytical approach, where H is the height of the injected OVA solution, L is the height of the total liquid, and M is the height of the slit center to the inner bottom of the cuvette (fixed at 6 mm in this study). The following assumptions were made: (i) diffusion follows Fick’s laws with a constant, concentration-independent diffusion coefficient during the experiment; (ii) one effective diffusion coefficient value is used to describe diffusion behaviors for the region of (0,H) and the region of (L-H,L), since H<<L; (iii) cross-diffusion caused by other species, such as HA and ions, is not taken into consideration; and (iv) a semi-infinite boundary is assumed, since almost no OVA molecules reached the boundary (L). The OVA concentration as a function of distance (x) and time (t) is shown in Eq. (1).

(1) C(x,t)=C04πDt∫0Hexp-(x-ε)2/4Dt+exp-(x+ε)2/4Dtdε

After integration, Eq. (2) is obtained.

(2) C(x,t)=C02erfH-x2Dt+erfH+x2Dt

where $C_{0}$ is the injected OVA concentration, and x is equal to M (fixed to 6 mm). For the initial condition, $0<x<H,\;C=C_{0}$; and x>H,C=0. For the boundary condition, $\frac{\partial C}{\partial x}(0,t)=0$. As noted, Eq. (2) is equivalent to Eq.2.15 listed in Crank’s classical book, The Mathematics of Diffusion.^26^ The best-fit value of the diffusion coefficient (D) was obtained by fitting the experimental data into the MATLAB (MathWorks, MA, USA)-programmed model.

### Numerical finite difference method

The second method models drug diffusion in one dimension and provides a numerical solution. Following the above assumptions (i), (ii), and (iii), with a finite difference method, the height of the total liquid (L) was equally divided into n discrete compartments (400 was chosen for n in this study), and diffusion behavior inside each compartment can be described by an ordinary differential equation. For 2≤i≤n-1, the concentration change $\left(\frac{dC_{i}}{dt}\right)$ is described by Eq. (3).

(3) $$\frac{dC_{i}}{dt}=D\frac{C_{i-1}\;-\;2C_{i}\;+\;C_{i+1}}{(L/n{)}^{2}}$$

For the 1st compartment, the concentration change $\left(\frac{dC_{1}}{dt}\right)$ is described by Eq. (4).

(4) $$\begin{array}{c} \frac{dC_{1}}{dt}=-D\frac{C_{1}\;-\;C_{2}}{(L/n{)}^{2}} \end{array}$$

For the $n^{\text{th}}$ compartment, the concentration change $\left(\frac{dC_{n}}{dt}\right)$ is described by Eq. (5).

(5) dCndt=DCn-1-Cn(L/n)2

About the initial condition, $C=C_{0}$ for $1\leq i\leq \frac{H}{L}\times n$ (round to the closest integer); and C=0 for the remaining compartments. By fitting the experimental data to the concentration of the compartment associated with the slit height, the best-fit value of the diffusion coefficient (D) was numerically solved using MATLAB (MathWorks, MA, USA).

### Numerical finite element method

The third method models drug diffusion in three dimensions using a finite element method in COMSOL Multiphysics^®^ (COMSOL, MA, USA). As shown in Fig. 1(d), the geometry of the cuvette is modeled, and the diffusion of OVA is governed by Fick’s second law: $\frac{\partial C}{\partial t}=D\frac{\partial^{2}C}{\partial x^{2}}$. The cuvette could be divided into four domains, from bottom to top: the domain representing the injected liquid (the bottom one in blue), the domain representing the slit region (the middle one in blue), and two other domains representing the regions below and above the slit. Regarding the initial condition, $C=C_{0}$ in the domain representing the injected liquid, and C=0 in other domains. While meshing all domains, the element size was set to “extremely fine” to achieve the highest resolution when solving the partial differential equation. The best-fit value of diffusion coefficient (D) was estimated by fitting the average concentration within the domain representing the slit region to experimental data using the BOBYQA algorithm.

### Rheological characterizations

For NaHA solutions, steady-shear rheology was performed on a Discovery HR-30 rotational rheometer (TA Instruments, DE, USA) fitted with a 40 mm stainless-steel parallel-plate geometry on a Peltier plate at a measurement gap of 0.300 mm. A solvent trap was used throughout to suppress evaporation. Each sample was equilibrated to the set temperature of 22.5 °C prior to measurement. Flow curves were acquired as a logarithmic shear-rate sweep from 10,000 to 0.01 s^−1^ at 5 points per decade. Steady-state sensing was enabled at every point: the viscosity was averaged over a 4 s sampling period, and a reading was accepted once 5 consecutive samples agreed to within a 2.5% tolerance. For the determination of the zero-shear viscosity, each flow curve was truncated at both extremes to retain only reliable data. At the low-shear end, points with a shear stress below about 0.2 1 Pa were discarded to keep the measurement above the torque-resolution limit of the instrument. At the high-shear end, data above approximately 2500 s^−1^ were excluded due to the measured normal force rapidly dropping, indicating edge/meniscus distortion from the high rotational speed rather than the true material response. The zero-shear viscosity, $\eta_{0}$, was then obtained by fitting the retained flow curve to the Carreau–Yasuda model.^27^

## Results and discussions

### Method validation and comparison

The diffusion of OVA in NaHA solutions with varying HA concentration and MW was investigated. Fig. 2 shows the results of one replicate of OVA diffusion in 1 mg/mL HA144K solution, including the concentration measured at the slit (scattered dots) and the curves with the best-fit diffusion coefficients obtained using the three methods. As shown, no OVA was detected at the initial stage until OVA molecules diffused upward into the slit region, after which the concentration increased continuously. All three methods fit the experimental data, as evidenced by the average R^2^ values across the whole data set: 0.9897 ± 0.0131 for analytical, 0.9890 ± 0.0134 for numerical (finite difference), and 0.9894 ± 0.0143 for numerical (finite element), indicating that modeling assumptions are acceptable. Similar diffusion coefficient values were obtained: compared to the analytical method, the values calculated by the numerical (finite difference) method are, on average, 3.1 ± 0.9% lower, and those calculated by the numerical (finite element) method are 6.2 ± 5.1% lower. The entire dataset of diffusion coefficient values is provided in Table S1 in the Supplementary Materials, and the values by the numerical (finite element) method were used for further analysis and plotting shown below.

The values obtained were also compared with previously reported values and with those measured by other techniques, as listed in Table 1. The measured diffusion coefficient of OVA in PBS was (7.05 ± 0.22) × 10^−7^ cm^2^/s, which differed less than 15% from other measured values in similar aqueous media and temperatures. The measured diffusion coefficients of OVA in diluted NaHA solutions were compared with those obtained by DLS. It is worth noting that DLS can be used to determine protein diffusion coefficients in diluted/semi-diluted polymer solutions, provided that multiple scattering is avoided and cross-diffusion is not considered.^15,28^ The detailed DLS measurement and data analysis are listed in Section S1 of the Supplementary Materials. As shown, our UV–vis spectrophotometer-based method produced diffusion coefficient values that were, on average, 3.5% lower than those from the DLS method, suggesting that both methods were capable and interchangeable for diffusion measurements in the HA solutions tested. However, our developed method can be applied to higher, more viscous NaHA solutions (e.g., 5 and 7.5 mg/mL), whereas the DLS method may raise concerns about multiple scattering, a non-continuous medium, and high viscosity. However, there may also be an upper limit to the medium viscosity for our method, as we found that while NaHA1670K increases beyond 5 mg/mL (corresponding to a viscosity of 1.29 Pa·s), the curve shape and diffusion coefficient values become more variable and noisier. Furthermore, the concentration ranges in the present study may be able to reflect the reported HA content in some biological fluids and tissues, e.g., ~ 2–3 mg/mL in the knee joint, ~ 0.2 mg/mL in the vitreous body, and ~ 400–500 *μ*g/g in skin.^29^ Nevertheless, the HA solutions can’t reproduce the full composition and structures of in vivo extracellular matrices.

### OVA diffusion coefficients in NAHA solutions

Fig. 3 presents the effective diffusion coefficients of OVA. For high-MW HA ranging from 144 to 1670 kDa, the diffusion coefficient decreases to a similar degree as the HA concentration increases. For example, at 5 mg/mL, the similar values with around 10% difference were observed: (5.12 ± 0.10) × 10^−7^ cm^2^/s for HA144K, (5.12 ± 0.31) × 10^−7^ cm^2^/s for HA337K, (5.69 ± 0.28) × 10^−7^ cm^2^/s for HA690K, and (5.41 ± 0.31) × 10^−7^ cm^2^/s for HA1670K, which were around 24% lower than that in PBS: (7.05 ± 0.22) × 10^−7^ cm^2^/s. For HA42K, which has an MW close to OVA (MW: 45 kDa), the value was (8.93 ± 0.48) × 10^−7^ cm^2^/s at 1 mg/mL, 27% higher than in PBS; and the diffusion coefficient decreased substantially at higher HA concentrations. For low-MW HAs, such as HA4.7 K, within the concentration range investigated, OVA diffusivity was slightly higher than PBS. These observations can be explained by the polymer obstruction effect theory^30^: within the concentration range being explored, high-MW HA chains can be regarded as immobilized networks, dramatically increasing the diffusion path length for OVA, whereas low-MW HA chains self-diffuse rapidly and do not act as an obstacle to hinder OVA diffusion or even slightly increase it. In addition, while HA MW is above 144 K, HA MW had no significant impact on OVA diffusivity, consistent with previous literature reporting that protein and polysaccharide diffusivities in dextran solutions were not affected by dextran MW.^31,32^ Beyond HA properties, other factors that can affect protein diffusion, including medium pH and ionic strength, as well as other excipients/chemicals, are worth investigating in future studies. Our findings also support the notion that cleaving HA into sufficiently small units (e.g., less than 4.7 kDa) using enzymatic (e.g., hyaluronidase) and non-enzymatic (e.g., hydrolysis, ultrasound, thermal) methods can enhance protein diffusion under in vivo and in vitro conditions.^33,11,34^

Furthermore, the OVA diffusion coefficients obtained in NaHA solutions with high MW (144 kDa and above) were averaged and evaluated using a set of diffusion models for polymer solutions. As shown in Table 2, all models provide a good fit for our data. The Phillies model^35^ gave an R^2^ value of 0.9880. In the equation, it was experimentally found that the parameter *α* depended on the diffusant’s hydrodynamic radius and/or MW, and the parameter v generally ranged from 0.5 (for high-MW diffusants) to 1 (for low-MW diffusants).^30,35^ Our best-fit value of v was 0.6591, indicating that OVA has an intermediate MW relative to HA networks. The Altenberger and Tirrel model^36^ provided an R^2^ value of 0.9870, and the parameter α was correlated with the diffusant hydrodynamic radius, and the parameter β was sourced from the interaction between the diffusant and the polymer.^30,36^ The model of Petit et al.^37^ could also fit the data (R^2^ value: 0.9877), where their model treated the medium as a transient network with an average mesh size, and the parameter α was a function of the diffusant molecule size and the polymer’s mesh size, and *v* was a constant dependent on the solvent.^30,37^ Furthermore, Wang’s model offered an R^2^ value of 0.9673, and the parameter α is related to diffusant geometry.^38^

### NaHA solution viscosity

Zero-shear viscosity $\left(\eta_{0}\right)$ of HA solutions was determined from the viscosity-shear rate profiles for six HA MWs at four concentrations (referred to as “c”), together with a PBS-only control $\left(\eta_{s}=9.41\times 10^{-4}\;Pa\cdot \;s\right)$. Specific viscosities were computed as $\eta_{sp}=\left(\eta_{0}-\eta_{s}\right)/\eta_{s}$. Plotting $\eta_{sp}$ against the dimensionless coil-overlap parameter c[η] (or $c/c^{*}$, the ratio of the concentration to the coil-overlap concentration) collapses the concentration series for all six MWs onto a single curve (as shown in Fig. 4).^39,40^ The data corroborate prior literature^41–43^ and resolve three distinct power-law regimes of the relationship between the specific viscosity and the coil-overlap parameter, $\eta_{sp}∼(c[\eta ]{)}^{n}$, with exponents that step upward at two crossovers.^44,45^ In the dilute regime where c[η]<1, HA coils are isolated from each other and contribute linearly to viscosity (n≈1.0). At concentrations above coil overlap $\left(c^{*}\right)$ where 1<c[η]<6, the onset of interchain contact causes the exponent to rise (n≈2.0), and a second crossover where c[η]>6 marks the entanglement concentration $c_{e}$, beyond which the exponent increases again (n≈4.3). The ratio $c_{e}/c^{*}\approx 6$ is within the expected range of entangled polymer solutions.^44,45^ Only the highest MWs at their higher concentrations populate the entangled regime: the 1670 kDa sample at ≥2.5 mg/mL and the 690 kDa sample at ≥5.0 mg/mL. In addition, the intrinsic viscosity [η], a measure of a polymer’s ability to increase solvent viscosity, was calculated, with the detailed information shown in Section S2 and Figure S1 in the Supplementary Materials.

The relationship between OVA’s diffusion coefficient and NaHA solution’s viscosity was also investigated. As shown in Fig. 5, the correlation deviates from the classical Stokes-Einstein equation $D=kT/6\pi \eta r_{h}$, where the diffusant’s diffusion coefficient (D) and solvent viscosity (η) should be inversely proportional. This observation can be explained by the fact that the Stokes-Einstein equation assumes that particles diffuse through a continuous solvent, whereas the distributions of HA polymers in the solvent introduce discontinuities in the medium. The HA polymer solution can be modeled as comprising polymer-unaffected and polymer-affected regions, in which OVA molecules exhibit different diffusion mechanisms, and the local viscosities (micro-viscosity) of these regions differ from the bulk viscosity (macro-viscosity) measured by a rheometer.^46–49^ It is also worth noting that the polymer-affected regions can be significantly larger than the volume of HA chains, because strong hydrogen bonds form between HA and water molecules, thereby altering the surrounding environment.^50–52^

## Conclusion

In this study, a straightforward UV–vis spectrophotometer-based method was developed to measure the diffusion coefficients of OVA in HA solutions. By combining a custom-designed cuvette with controlled sample loading and mathematical modeling, diffusion coefficients could be obtained by fitting the experimental data. Furthermore, it was found that high-MW HA reduced OVA diffusivity in a HA concentration-dependent manner, whereas low-MW HA produced minimal obstruction and either did not change or even slightly enhanced diffusion. Overall, this developed method offers several advantages, including accurate and reproducible measurements, material sparing, low cost, and instrument accessibility, and can therefore serve as an alternative to more specialized diffusion measurement techniques. The present work also provides a better understanding of protein diffusion within the extracellular matrix and offers insights into protein molecule/formulation design.

## Supplementary Material

2

Supplementary material associated with this article can be found in the online version at doi:10.1016/j.xphs.2026.104507.

## Figures and Tables

**Fig. 1.**
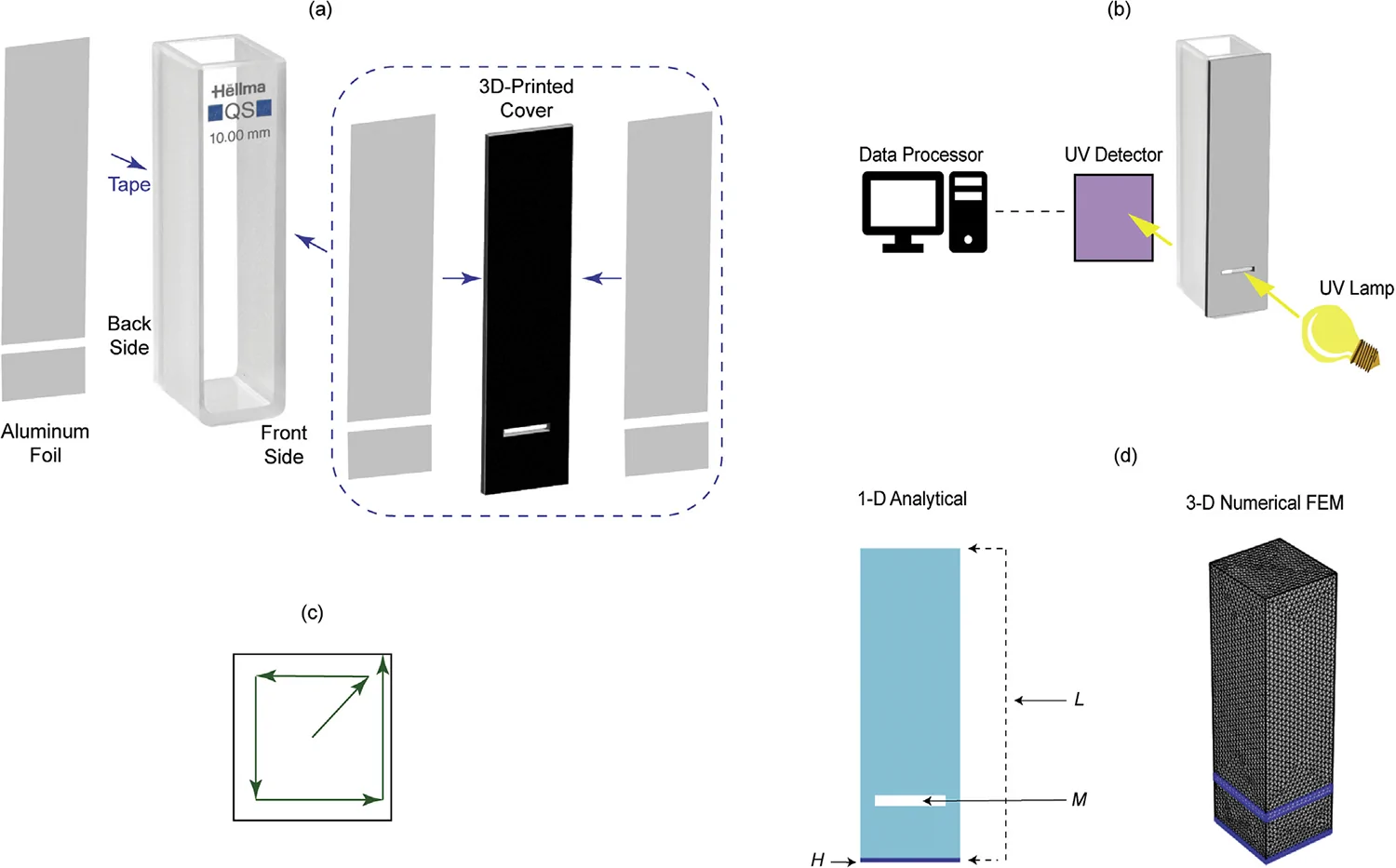
Cartoons of the experimental setup for diffusion coefficient measurements. (a) Custom-designed cuvette modified from a standard UV cuvette installed with a 3D-printed cover with an open slit. (b) Illustration describing protein absorbance measured at the slit by a UV–vis spectrophotometer. (c) Protein sample injection path. (d) Diffusion model: 1-D for analytical method and 3-D for numerical (finite element) method.

**Fig. 2.**
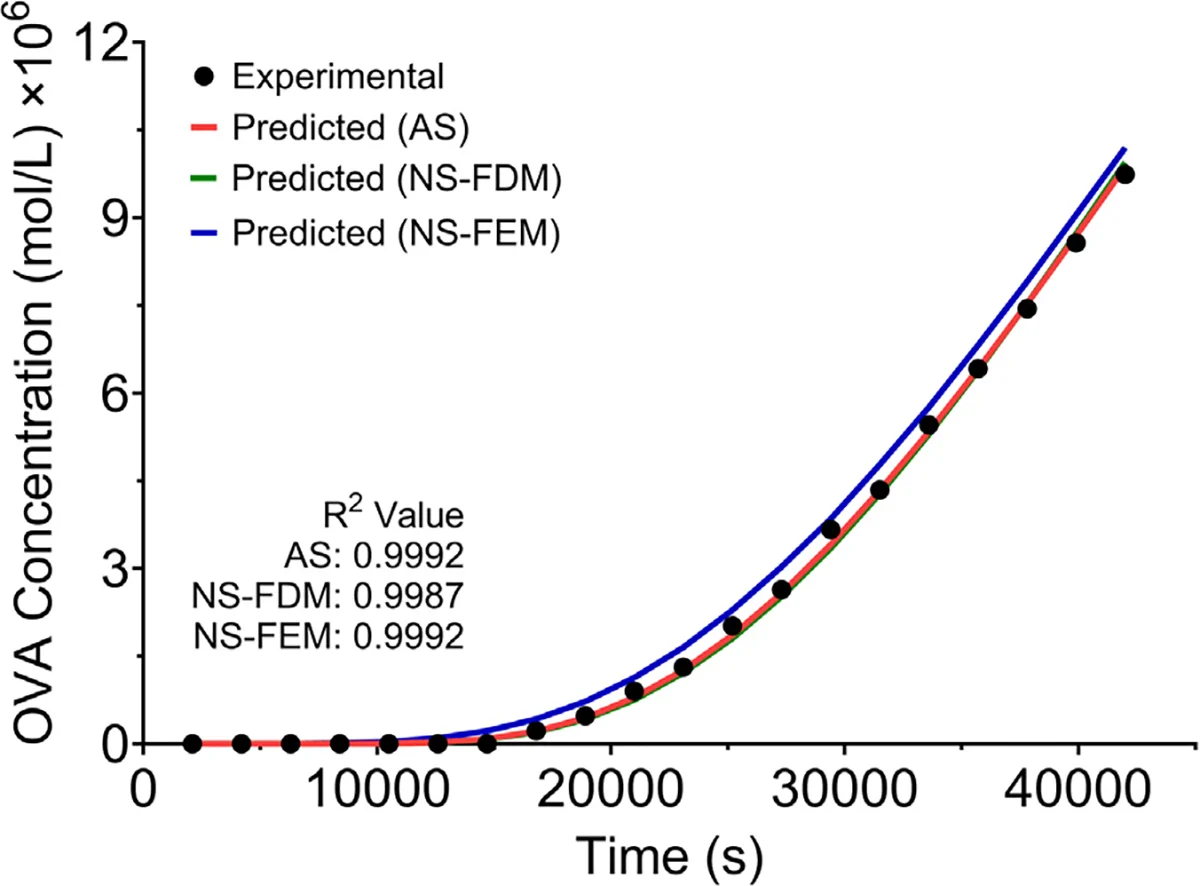
One example run of the concentration vs. time curve of OVA diffusion in 1 mg/mL NaHA (HA144K) solution. Note: the green curve overlaps with the red curve.

**Fig. 3.**
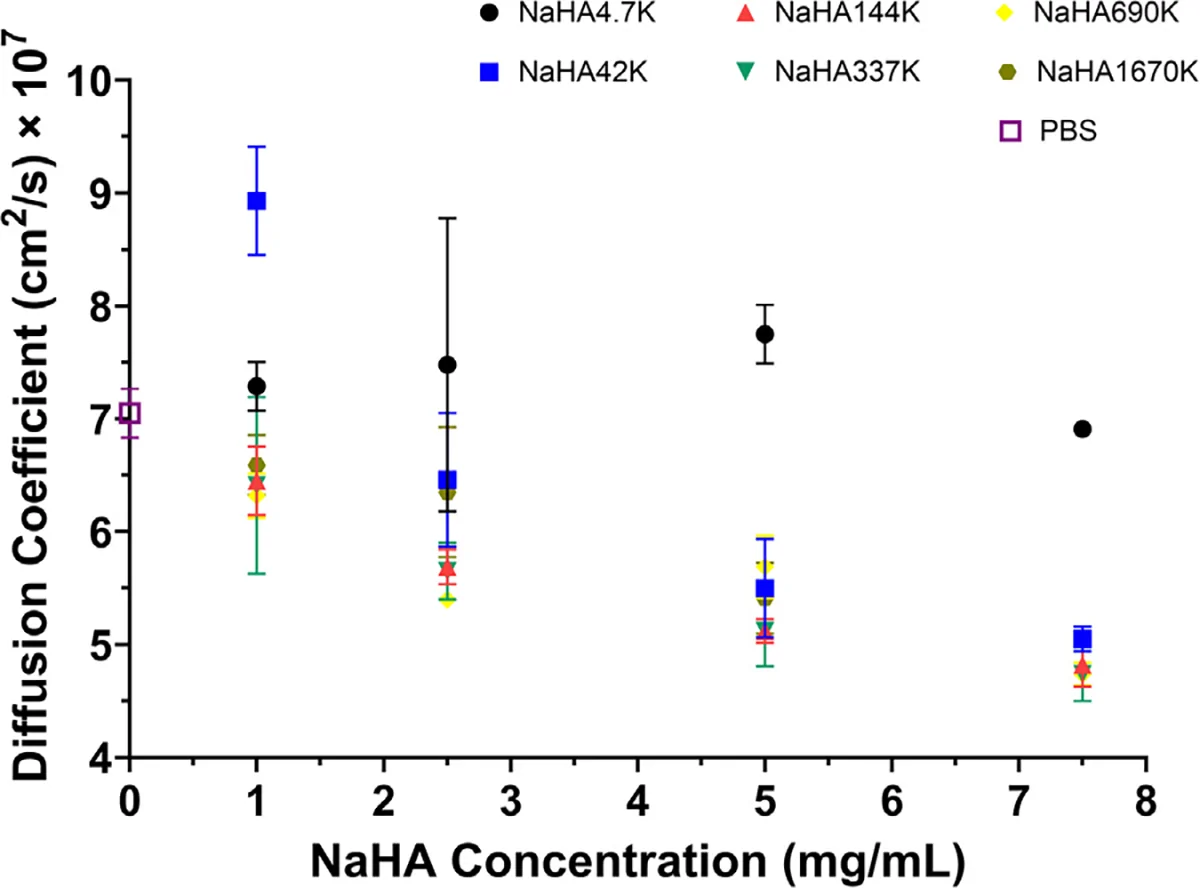
Diffusion coefficients of OVA in NaHA solutions with different HA MWs and concentrations.

**Fig. 4.**
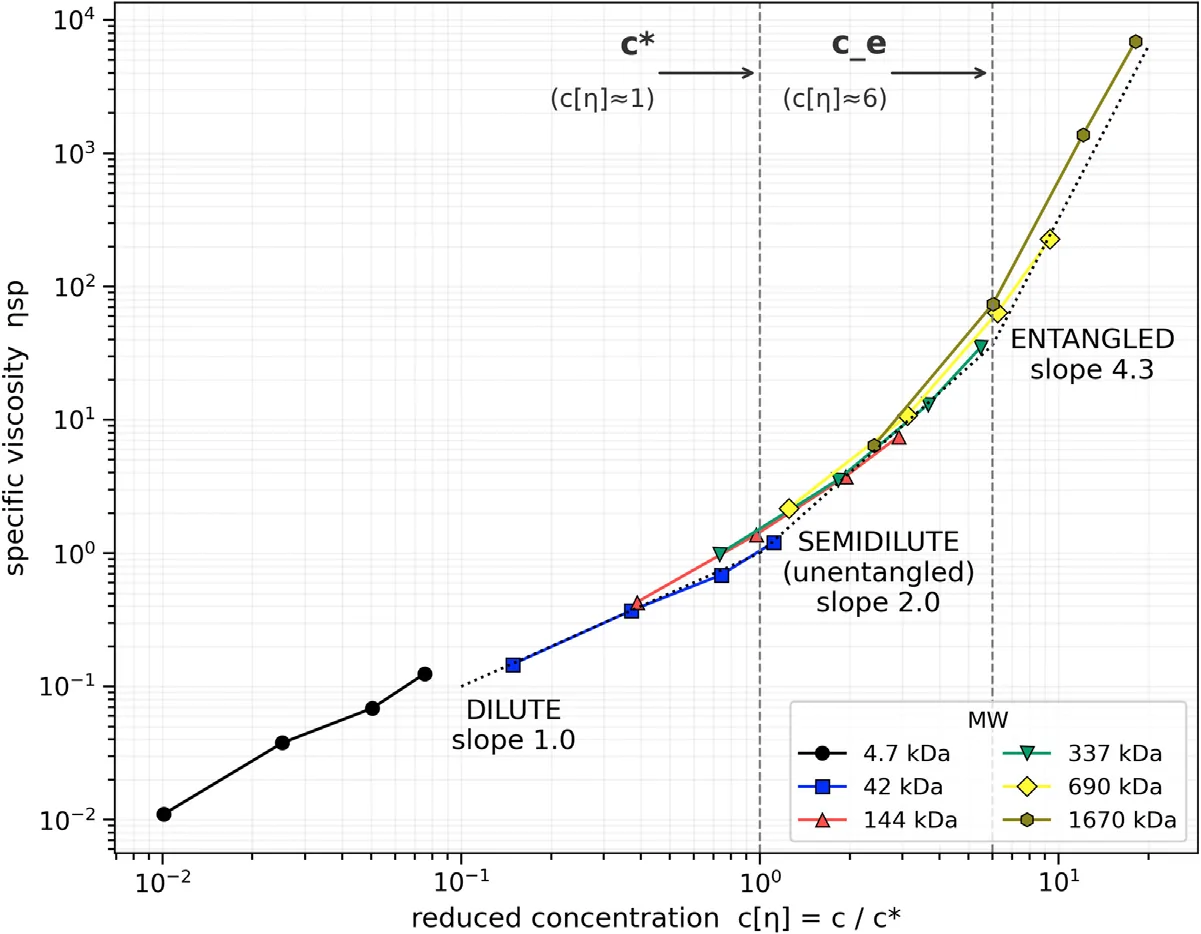
Specific viscosity of all six NaHA MWs plotted by their reduced concentration c[η]. Sloped dotted lines represent power law relationships with exponents 1.0, 2.0, and 4.3 for the dilute, semi-dilute, and entangled regimes, respectively; dashed vertical lines designate the overlap (c*) and entanglement (ce) crossovers at c[η]≈1 and ≈ 6, respectively.

**Fig. 5.**
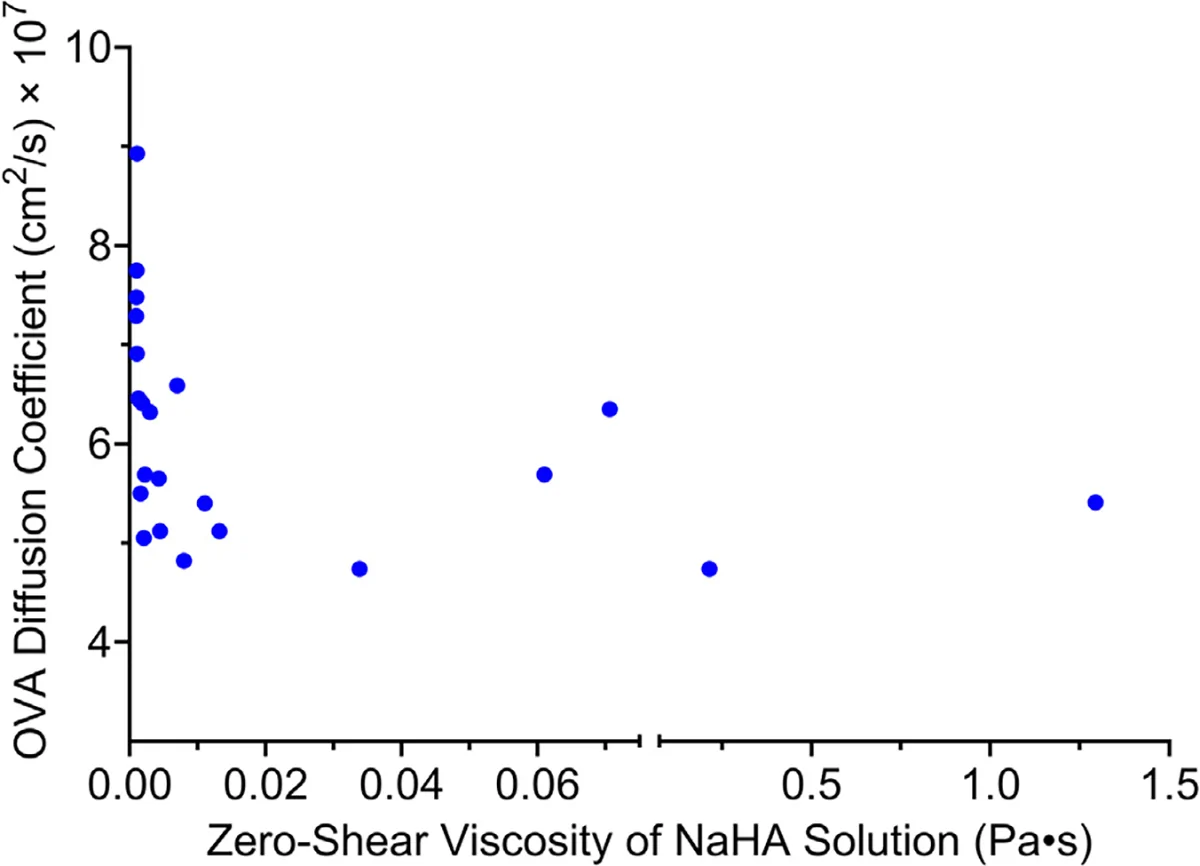
Plot of diffusion coefficient of OVA and zero-shear viscosity of the corresponding NaHA solutions.

**Table 1:** Diffusion coefficients obtained using our developed method, compared with those reported in the literature and from other methods.

Medium	Measured effective diffusion coefficient(10^−7^ cm^2^/s)	
	Our UV–vis spectrophotometer-based method	Other methods and/or literature reported values

PBS	7.05 ± 0.22 (22 °C)	6.0 to 7.75^15,25,53–56^
HA690K (1 mg/mL) in PBS	6.32 ± 0.20 (22 °C)	6.97 ± 0.35 (DLS, 22 °C)
HA690K (2.5 mg/mL) in PBS	5.40 ± 0.04 (22 °C)	5.25 ± 0.51 (DLS, 22 °C)
HA1670K (1 mg/mL) in PBS	6.59 ± 0.27 (22 °C)	6.79 ± 1.58 (DLS, 22 °C)
HA1670K (2.5 mg/mL) in PBS	6.35 ± 0.58 (22 °C)	6.57 ± 0.44 (DLS, 22 °C)

**Table 2:** Experimental diffusion coefficients fit with diffusion models.

Model	Reference	Best-fitting parameter	R^2^ Value

*D/D*_0_ = exp (−*αC*^0.5^)	Cukier^57^	*α* = 4.052	0.9438
*D/D*_0_ = exp (−*αC*^*v*^)	Phillies^35^	*α* = 9.531; *v* = 0.6591	0.9880
*D/D*_0_ = 1 – *αC*^0.5^ – *βC*	Altenberger and Tirrel^36^	*α* = 2.837; *β* = −9.607	0.9870
*D/D*_0_ = 1/(1 + *αC*^2*v*^)	Petit et al.^37^	*α* = 16.86; *v* = 0.3686	0.9877
*D/D*_0_ = 1 – *α*∅	Wang^38^	*α* = 48.04	0.9673

*C*: NaHA concentration in g/mL.

∅: NaHAvolume fraction between 0 and 1.

## Data Availability

Data presented in this work are available from the corresponding author upon request.
