# Supplementary material for: Development of a method to measure protein diffusivities in hyaluronic acid solutions based on a UV–vis spectrophotometer
Source: J Pharm Sci. Author manuscript; Available in PMC 2026 Nov 5. (PMC13739415; doi:10.1016/j.xphs.2026.104507)
Supplement: Supplementary file 1 [file NIHMS2213316-supplement-2.docx]

Supplementary Materials for the Manuscript Entitled

**Development of a method to measure protein diffusivities in hyaluronic acid solutions based on a UV-Vis spectrophotometer**

Hao Lou,^a,*^ Meng-Juan Pang,^a^ Benjamin Southard,^a,b^ Hashim M. Reza^a^

^a^ Department of Pharmaceutical Chemistry, The University of Kansas, Lawrence, Kansas 66047, USA

^b^ Biopharmaceutical Innovation & Optimization Center, The University of Kansas, Lawrence, Kansas 66047, USA

^*^ Correspondence to: lou0@ku.edu

| Table S1. Diffusion coefficients of OVA in HA solutions. | | | | |
| --- | --- | --- | --- | --- |
| NaHA Concentration (mg/mL) | HA MW (average) | Diffusion Coefficient (cm^2^/s) × 10^7^ at 22°C (n=3) | | |
|  |  | Analytical | Numerical (Finite Difference) | Numerical (Finite Element) |
| 0 | PBS only | 7.62 ± 0.25 | 7.36 ± 0.23 | 7.05 ± 0.22 |
| 1 | 4.7 kDa | 8.15 ± 0.25 | 7.62 ± 0.22 | 7.29 ± 0.22 |
| 2.5 |  | 9.16 ± 0.44 | 8.81 ± 0.41 | 7.48 ± 1.30 |
| 5 |  | 7.90 ± 0.23 | 7.85 ± 0.24 | 7.75 ± 0.26 |
| 7.5 |  | 7.32 ± 0.19 | 7.15 ± 0.31 | 6.91 ± 0.08 |
| 1 | 42 kDa | 8.06 ± 0.15 | 7.78 ± 0.14 | 8.93 ± 0.48 |
| 2.5 |  | 6.90 ± 0.39 | 6.67 ± 0.37 | 6.46 ± 0.59 |
| 5 |  | 6.00 ± 0.57 | 5.82 ± 0.54 | 5.50 ± 0.44 |
| 7.5 |  | 5.42 ± 0.18 | 5.27 ± 0.17 | 5.05 ± 0.11 |
| 1 | 144 kDa | 6.91 ± 0.21 | 6.68 ± 0.20 | 6.45 ± 0.30 |
| 2.5 |  | 6.26 ± 0.16 | 6.06 ± 0.16 | 5.69 ± 0.15 |
| 5 |  | 5.53 ± 0.16 | 5.37 ± 0.16 | 5.12 ± 0.10 |
| 7.5 |  | 4.96 ± 0.40 | 4.83 ± 0.38 | 4.82 ± 0.19 |
| 1 | 337 kDa | 6.83 ± 0.57 | 6.60 ± 0.54 | 6.41 ± 0.78 |
| 2.5 |  | 6.22 ± 0.28 | 6.02 ± 0.27 | 5.65 ± 0.25 |
| 5 |  | 5.49 ± 0.50 | 5.34 ± 0.48 | 5.12 ± 0.31 |
| 7.5 |  | 4.74 ± 0.54 | 4.61 ± 0.53 | 4.74 ± 0.24 |
| 1 | 690 kDa | 6.82 ± 0.15 | 6.60 ± 0.14 | 6.32 ± 0.20 |
| 2.5 |  | 5.94 ± 0.05 | 5.76 ± 0.04 | 5.40 ± 0.04 |
| 5 |  | 6.24 ± 0.30 | 6.05 ± 0.29 | 5.69 ± 0.28 |
| 7.5 |  | 4.79 ± 0.26 | 4.66 ± 0.25 | 4.74 ± 0.10 |
| 1 | 1670 kDa | 7.01 ± 0.18 | 6.78 ± 0.17 | 6.59 ± 0.27 |
| 2.5 |  | 6.80 ± 0.46 | 6.58 ± 0.44 | 6.35 ± 0.58 |
| 5 |  | 5.92 ± 0.39 | 5.74 ± 0.37 | 5.41 ± 0.31 |

Section S1: Dynamic Light Scattering (DLS) to Determine OVA Diffusion Coefficients

The diffusivities of OVA in HA solutions were analyzed using a Malvern Zetasizer® (Malvern Instruments, Malvern, UK) with a 633 nm laser at a 173° scattering angle with backscatter detection only at 22°C. Measurements were performed in 9 replicates per condition.

A normalized second-order autocorrelation function (ACF) curve ($G^{2}\left( t \right)-1$) against time was obtained from the DLS measurements and then fit to an equation containing three exponential terms, as shown in Eq. S1.

$G^{2}\left( t \right)-1=\left( I_{f1}exp\left( -t/\tau_{f1} \right)+I_{f2}exp\left( -t/\tau_{f2} \right)+I_{s}{exp\left( -t/\tau_{se} \right)}^{\beta} \right)^{2}$	(Eq. S1)

where the first two terms were related to the diffusivities of OVA and HA (no consideration of cross diffusion), and the last term reflected HA chain slow relaxation and imperfect signal collections. Given the best-fitted value of $\tau_{f}$, diffusion coefficients (*D*) of OVA and HA were determined by the equation $D=1/q^{2}\tau_{f}$, with $q=\frac{4\pi n_{0}}{\lambda}sin(\frac{\theta}{2})$, where n_0_ was solvent refractive index, 𝜆 was the wavelength of incident light, and 𝜃 was the angle of incident light.

Section S2: Determination of Intrinsic Viscosity

For each HA MW, the intrinsic viscosity [η] was determined using one of two approaches, depending on the MW, and plotted in Figure S1. For the 144, 337, 690, and 1670 kDa samples, [η] was assigned from the Mark–Houwink–Sakurada (MHS) relation reported by Di Meo et al. for pharmaceutical-grade NaHA, [η] = 5.5x10^-4^·M^0.75^ (dL/g).^1^ These MWs fall within the 56-2500 kDa range over which that relation was established, and our flow curves did not extend to concentrations far enough below the coil-overlap concentration (c*) to permit an independent extrapolation of [η] for these samples. For the 4.7 and 42 kDa samples, which lie below the MW range of Di Meo’s MHS relation, [η] was instead determined directly. Three or more concentrations for each fell below c*, allowing the reduced viscosity (η_sp_/c) to be extrapolated to infinite dilution by the Huggins method, yielding [η] = 0.10 and 1.48 dL/g, respectively.^2^ The 42 kDa sample sits just below the MHS calibration range, and its independently extrapolated [η] (1.48 dL/g) agrees with the value predicted by Di Meo’s MHS relation (1.55 dL/g) to within 5%, supporting its use for the higher-MW samples. The 4.7 kDa HA oligomer is a special case. Since this chain spans only a few persistence lengths, it cannot adopt a random-coil conformation, so the coil-based scaling that underlies the MHS relation does not apply;^3^ its Huggins [η] is reported as an empirical hydrodynamic descriptor only.

Figure S1. Mark–Houwink plot showing intrinsic viscosity of NaHA solutions plotted by molecular weight. The 4.7 and 42 kDa points are fit by extrapolation from their dilute concentrations. The 144-1670 kDa data points are calculated from the MHS relation fit (grey line) from Di Meo et al.^1^ (data shown in grey circles)

**References**

(1) Di Meo, C.; Stellavato, A.; d'Agostino, M.; D'Agostino, A.; Schiraldi, C.; La Gatta, A. Hyaluronan size and concentration: Effect on key biophysical and biochemical features. *International Journal of Biological Macromolecules* **2024**, *282*, 137125. DOI: <https://doi.org/10.1016/j.ijbiomac.2024.137125>.

(2) Huggins, M. L. The Viscosity of Dilute Solutions of Long-Chain Molecules. IV. Dependence on Concentration. *Journal of the American Chemical Society* **1942**, *64* (11), 2716–2718. DOI: 10.1021/ja01263a056.

(3) Cleland, R. L. The persistence length of hyaluronic acid: An estimate from small-angle X-ray scattering and intrinsic viscosity. *Archives of Biochemistry and Biophysics* **1977**, *180* (1), 57–68. DOI: <https://doi.org/10.1016/0003-9861(77)90008-X>.
